# Skeletal Muscle Pathophysiology: The Emerging Role of Spermine Oxidase and Spermidine

**DOI:** 10.3390/medsci6010014

**Published:** 2018-02-14

**Authors:** Manuela Cervelli, Alessia Leonetti, Guglielmo Duranti, Stefania Sabatini, Roberta Ceci, Paolo Mariottini

**Affiliations:** 1 Department of Science, Università degli Studi di Roma “Roma Tre”, 00146 Rome, Italy; alessia.leonetti@uniroma3.it (A.L.); paolo.mariottini@uniroma3.it (P.M.); 2 Department of of Movement Human and Health Sciences, Unit of Biology, Genetics and Biochemistry, Università degli Studi di Roma “Foro Italico”, Piazza Lauro De Bosis 15, 00135 Rome, Italy; guglielmo.duranti@uniroma4.it (G.D.); stefania.sabatini@uniroma4.it (S.S.); roberta.ceci@uniroma4.it (R.C.)

**Keywords:** aging, atrophy, autophagy, oxidative stress, polyamines, skeletal muscle, spermidine, spermine oxidase, transgenic mouse

## Abstract

Skeletal muscle comprises approximately 40% of the total body mass. Preserving muscle health and function is essential for the entire body in order to counteract chronic diseases such as type II diabetes, cardiovascular diseases, and cancer. Prolonged physical inactivity, particularly among the elderly, causes muscle atrophy, a pathological state with adverse outcomes such as poor quality of life, physical disability, and high mortality. In murine skeletal muscle C2C12 cells, increased expression of the spermine oxidase (SMOX) enzyme has been found during cell differentiation. Notably, SMOX overexpression increases muscle fiber size, while SMOX reduction was enough to induce muscle atrophy in multiple murine models. Of note, the SMOX reaction product spermidine appears to be involved in skeletal muscle atrophy/hypertrophy. It is effective in reactivating autophagy, ameliorating the myopathic defects of collagen VI-null mice. Moreover, spermidine treatment, if combined with exercise, can affect D-gal-induced aging-related skeletal muscle atrophy. This review hypothesizes a role for SMOX during skeletal muscle differentiation and outlines its role and that of spermidine in muscle atrophy. The identification of new molecular pathways involved in the maintenance of skeletal muscle health could be beneficial in developing novel therapeutic lead compounds to treat muscle atrophy.

## 1. Introduction 

Skeletal muscle is the largest tissue of the human body and represents 40–50% of body weight, varying according to physiological and pathological conditions [1]. Skeletal muscle atrophy is a frequent and disabling condition. It involves different molecular mechanisms that occur during pathogenesis, hence knowledge of the cellular signal pathways that mediate muscle atrophy is still limited [2].

**Figure 1:**
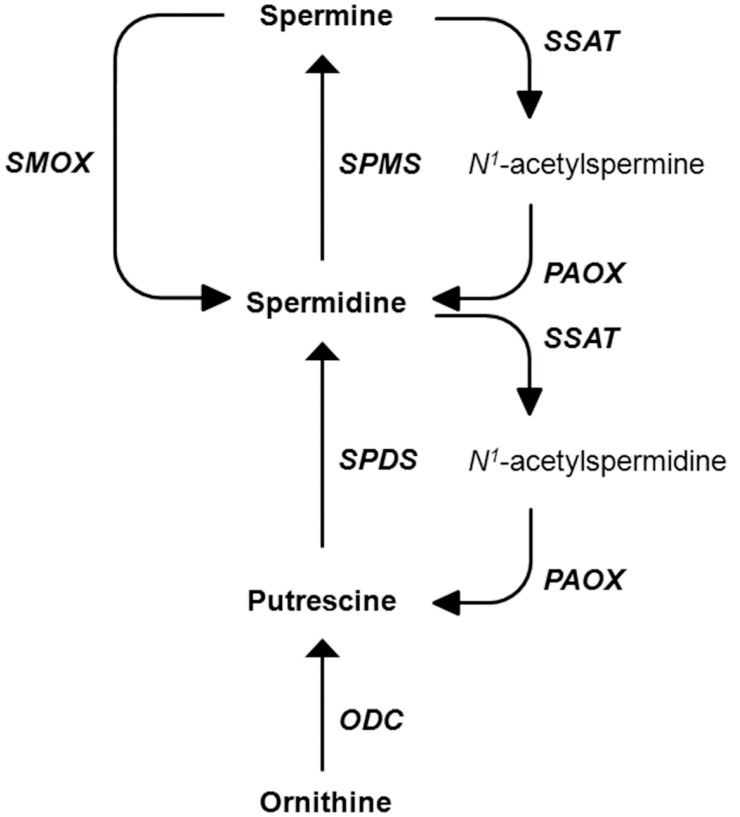
Polyamine metabolism. Schematic representation of mammalian polyamine metabolism showing enzyme network and substrate interconversion pathways. ODC: ornithine decarboxylase; SSAT: spermidine/spermine *N*^1^-acetyltransferase; PAOX: polyamine oxidase; SMOX: spermine oxidase; SPMS: spermine synthase; SPDS: spermidine synthase.

Polyamines (PAs) are essential for normal cell growth, proliferation, and differentiation, and the tissue levels of individual PAs are maintained and buffered via complex regulatory mechanisms [3,4]. The functional roles of the natural PAs, putrescine (Put), spermidine (Spd), and spermine (Spm), are under active investigation in broad research areas, from neuroscience to cancer and biochemistry [5,6,7,8,9]. Two key enzymes, ornithine decarboxylase and *S*-adenosylmethionine decarboxylase, control PA biosynthesis. PA catabolism is finely regulated by the enzymes *N*^1^-acetyltransferase, polyamine oxidase, and spermine oxidase (SMOX) (Figure 1).

Polyamine levels can be altered by physiological stimuli or by inhibitors or inducers of PA metabolic enzymes. Animal models with genetically altered PA synthesis or catabolism offer a versatile way to affect PA homoeostasis in different tissues and for a prolonged period. These models help to define the physiological importance of PAs by offering tools to develop treatment therapies for pathophysiological conditions derived from deregulated PA homeostasis [10]. Given the role of PAs in the development of many tissues, numerous studies have examined the relation between PAs and skeletal muscle atrophy and hypertrophy. The importance of PAs in muscle disease is highlighted by an alteration of their levels in muscular fibers undergoing degeneration and regeneration [10]. Altered PA levels are present in muscles of patients suffering from Duchenne muscular dystrophy, a pathology characterized by muscle fiber atrophy [11]. Limb girdle dystrophy patients, who display weakness and wasting of the muscles in the arms and legs, show higher levels of PAs in skeletal muscle [11,12]. Similarly, in mouse and hamster models of muscle dystrophy, PA content is altered compared to control muscles [12]. Although the strong association between PA levels and muscle mass is evident, the potential mechanism by which PAs regulate muscle growth is still unclear [10]. This review focuses on the role of SMOX in skeletal muscle pathophysiology, underlining its role in myogenesis and muscle atrophy. The use of a Total-SMOX animal model could help to find new therapies able to counteract physiological atrophy due to aging and pathology.

## 2. Skeletal Muscle Differentiation 

**Figure 2:**
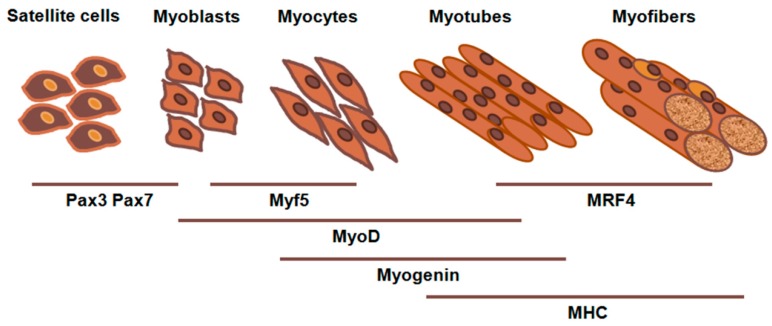
Schematic illustration of the skeletal muscle differentiation process. During myogenesis, Pax3 and Pax7 are activated in quiescent progenitors. Then, progenitor cells differentiate into proliferating muscle precursor cells (myoblasts) and Myf5, MyoD, and myogenin expression stimulates myoblasts to differentiate into myotubes. The terminal stage of differentiation is mediated by the activation of genes responsible for muscle fiber (myofiber) architecture and functionality such as MHCs. Pax: paired box; Myf5: myogenic factor 5; MyoD: myogenic factor 3; MRF4: myogenic regulatory factor 4; MHC: myosin heavy chain.

Skeletal muscle differentiation (myogenesis) is a finely regulated process involving a cascade of muscle-specific genes whose expression is coordinated in a timely manner to cell cycle withdrawal and synthesis of muscle contractile proteins. During embryogenesis, muscle fibers are established through a highly ordered multistep process leading from mononucleated-undifferentiated cells (myoblasts) to polynuclear cells (myotubes) [13]. The process is predominantly regulated by myogenic regulatory factors (MRFs) of the basic helix-loop-helix family of transcription factors including MyoD, Myf5, myogenin, and MRF4, together with other transcription factors such as paired box 3 (Pax3) and paired box 7 (Pax7) [14]. MyoD and Myf5, expressed before the onset of myogenic differentiation, promote proliferation and differentiation of myogenic progenitor cells into myoblasts [15], while myogenin plays an important role in the differentiation of myoblasts into myotubes and MRF4 participates in differentiation and cell fate determination [16] (Figure 2). 

It must be pointed out that exiting from the cell cycle is a critical regulatory event for successful myogenic differentiation. The elevation of MyoD expression in proliferating myoblasts induces transcriptional upregulation of cell-cycle inhibitors, such as the cyclin-dependent kinase inhibitor p21, that play a fundamental role in establishing the post-mitotic state in skeletal muscle, completing the differentiation process [17,18]. After cell cycle arrest, late differentiation markers such as myosin heavy chain (MHC) are induced [19] (Figure 2).

During adulthood, constant muscle remodeling is possible due to the presence of a specialized population of myogenic progenitors, the satellite cells, placed underneath the myofiber basal lamina. These cells, mitotically quiescent, act as muscle stem cells, allowing the repair and maintenance of myofibers. Upon damage or stress, satellite cells divide asymmetrically: some of them reconstitute the pool of quiescent satellite stem cells and others differentiate into myoblasts. Finally, they fuse together to form new myotubes or fuse with damaged myotubes to repair them [19]. The mouse C2C12 muscle cell line is a useful model system to study the differentiation process. In vitro, myogenic differentiation can be obtained by serum deprivation from myoblast cultures. In high serum, myoblasts proliferate, while after serum removal, they go into an early differentiation stage. Later, cells begin to fuse, forming multinucleated myotubes positive for the characteristic muscle-specific protein MHC [20,21,22]. 

Many molecules are highly regulated during the phenotypic conversion of rapidly dividing C2C12 myoblasts into fully differentiated post-mitotic myotubes. Among them, SMOX, the most recently characterized polyamine catabolic enzyme [23], has been shown to be modulated during C2C12 differentiation [24]. 

## 3. Spermine Oxidase and Muscle Tissue 

In animal cells, SMOX can be considered a multitasking enzyme, primarily involved in controlling PA metabolism, and its substrate Spm and reaction product Spd are ubiquitous polycations that have several important control functions in cells, ranging from basic DNA synthesis to regulation of cell proliferation and differentiation [25]. 

The SMOX gene is highly expressed in muscle tissue, as demonstrated by Cervelli et al. [26], who reported a high level of both transcript and enzymatic activity. Interestingly, in C2C12 myoblast cultures, after serum removal, SMOX messenger RNA (mRNA) activity was downregulated in the early stage of differentiation. Successively, SMOX was induced in a time-dependent manner, at the level of both transcription and enzymatic activity. Overall, the pattern of SMOX expression profile in the C2C12 muscle cell line showed high levels in proliferating myoblasts followed by low levels in differentiating myoblasts and high levels again in myocytes/myotubes [24]. 

The relatively high expression levels of SMOX in proliferating cells could promote cell expansion and/or survival. It is known that SMOX can be rapidly induced in response to stress and is responsible for H_2_O_2_ production in different cell lines and malignant tissues [27]. Thus, a decrease in its activity, and possibly a reduction in the level of H_2_O_2_, could allow for subsequent cell differentiation, with fully differentiated cells resuming its expression and activity. A failure of SMOX downregulation might result in defective differentiation and potentially contribute to neoplastic transformation. It is interesting that SMOX expression levels are high in several tumor cell types [28,29]. Hence, there needs to be investigation of mechanisms through which SMOX expression can be re-established, as occurs at the end of the differentiation process. Interestingly, a recent study suggested that muscle atrophy induced by limb immobilization, fasting, muscle denervation, and aging could be related to a significant reduction of SMOX expression, while on the other hand, when SMOX was overexpressed, muscle fiber size increased. Furthermore, SMOX overexpression resulted in a decrease of the genes that promote muscle atrophy and, conversely, an increase in the expression of genes that help to maintain muscle mass [30]. Overall, these results highlight the importance of SMOX in the pathophysiology of skeletal muscle. Remarkably, involvement of p21 has been observed in atrophy conditions: the induction of p21 actively promotes muscle atrophy, leading to reduced SMOX expression; on the other hand, a relatively low level of p21 allows a higher level of SMOX expression, which helps to maintain basal skeletal muscle gene expression and fiber size [30]. It has been reported that p21 plays an important role in the in vivo healing process in muscular injury [31], and moreover, it has been shown that p21 protein is modulated during C2C12 differentiation: it increases at cell cycle exit and entrance into differentiation and declines in a time-dependent manner thereafter [32]. 

**Figure 3:**
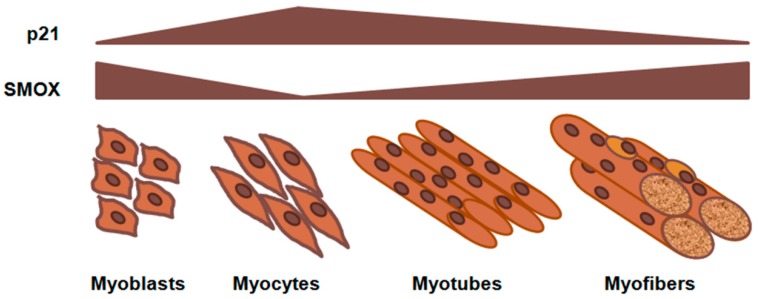
Hypothetical representation of SMOX regulation during myogenesis. During the early stage of muscle differentiation, p21 expression increases, leading to reduced SMOX expression. During the differentiation process, p21 declines in a time-dependent manner, allowing SMOX expression.

In Figure 3, a hypothetical scheme shows the possible relation between SMOX and p21 during myogenesis.

**Figure 4:**
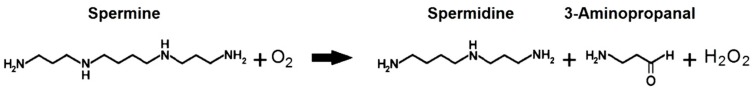
Enzymatic reaction catalyzed by SMOX. The physiological substrate spermine (Spm) is oxidized by the SMOX enzyme into spermidine (Spd) with the production of 3-aminopropanal and hydrogen peroxide.

Spermine oxidase catalyzes the direct back-conversion of Spm to Spd, 3-aminopropanal (3-AP), which is non-enzymatically converted to acrolein, and hydrogen peroxide (H_2_O_2_) (Figure 4), and each of these products can affect muscle tissue in various ways, as described in the following sections.

## 4. Hydrogen Peroxide and Muscle Tissue 

The reactive oxygen species (ROS) H_2_O_2_ produced by SMOX is a two-electron, nonradical oxidant molecule that is stable and freely diffuses within and between cells, and therefore acts as a signaling molecule. Numerous studies on myogenesis have indicated that muscle development is particularly sensitive to environmental and endogenous H_2_O_2_ levels. On the whole, H_2_O_2_ is directly engaged in modulating the expression of several enzymes related to the cellular redox state and plays a wide range of important roles in a variety of cells in a concentration-dependent manner [33,34,35,36]. It is to be noted that at low concentrations, H_2_O_2_ can activate various enzymes, such as phosphatases, modulating cell signaling; on the other hand, at high concentrations, it causes oxidative stress, leading to irreversible cell damage [37]. 

Some studies, conducted by treating cells with H_2_O_2_ exogenously, provide evidence for its negative effect on myoblast differentiation. Treatment with H_2_O_2_ slowed differentiation [38] and reduced myogenin and MHC protein content and creatine kinase activity, as well as troponin I gene transcription, in a dose-dependent manner [39]. The inhibition of myotube formation was reversible when the powerful antioxidant *N*-acetylcysteine had been previously added to the culture medium [39]. It was also reported that H_2_O_2_ administration markedly reduced *Myf5*, *MRF4* gene, and myogenin expression [40]. Another study showed that during the early stages of C2C12 myoblast differentiation, mildly toxic treatment with H_2_O_2_ resulted in the depletion of glutathione (GSH), the main thiol antioxidant, leading to further intracellular accumulation of ROS. The oxidative environment favored the activation of nuclear factor-kappa B (NF-κB), a redox-sensitive transcription factor, thus contributing to the lower expression of MyoD and impaired myogenesis [41]. 

On the other hand, other studies have shown a crucial role for endogenous ROS concentrations during skeletal muscle differentiation. It has been reported that an increase in skeletal muscle NADPH oxidase isoform 2 (NOX2) activity during differentiation leads to a rise in superoxide anion O_2_^−^, a molecule quickly converted by dismutation into H_2_O_2_. In line with the positive role of ROS, this increase seems to be crucial to muscle differentiation via NF-κB/inducible nitric oxide synthase (iNOS) pathway activation [42] and to the promotion of skeletal muscle precursor cell proliferation [43]. Furthermore, it has been observed that enhancement of the endogenous H_2_O_2_ level can regulate the cellular GSH redox balance from the very early stage to the fully differentiated myotube formation stage [44]. On the whole, these data clearly show that myogenesis is a process very sensitive to the intracellular redox environment, and at the same time supports the notion that H_2_O_2_ can have different outcomes depending on its intracellular concentration.

## 5. Acrolein and Muscle Tissue 

Numerous conditions, such as oxidative stress, inflammation, alcoholic myopathy, and renal failure in which acrolein is increased, have been associated with muscle deterioration and dysfunction, resulting in disease. Moreover, increased muscle catabolism has been linked to an exogenous source of acrolein due to cigarette smoking [45]. Exposure of skeletal myotubes to cigarette smoke stimulates muscle catabolism via increased oxidative stress, activation of p38 mitogen-activated protein kinases (p38MAPK), and upregulation of muscle-specific E3 ubiquitin ligases [45]. In addition, acrolein treatment was sufficient to induce an increase of free radicals, activation of p38 MAPK, up-regulation of the muscle-specific E3 ligases atrogin-1 and MuRF1, degradation of MHC, and atrophy of myotubes. Inhibition of p38MAPK by SB203580 abolished acrolein-induced muscle catabolism [46]. These studies demonstrated that acrolein is able to activate a signaling cascade, inducing muscle catabolism in skeletal myotubes. Acrolein can also react with amino acid residues in proteins, consequently modifying protein function and inducing apoptosis [47] or tissue damage such as brain infarction [48,49]. Notwithstanding that high levels of acrolein within cells have been linked to toxicity, it has recently been demonstrated that SMOX plays a central role in the formation of bile canalicular lumen in liver cells by activating the protein kinase B (AKT) pathway through acrolein production [50]. 

## 6. Spermidine and Muscle Tissue 

Considering the new emerging role of SMOX in muscle physiology, it is of primary importance to have full knowledge of this enzyme and the role of its catabolic product Spd. In several model systems such as yeast, flies, worms, and human immune cells, Spd levels decrease during aging, and by giving Spd as a dietary supplement, lifespan is extended [51]. In fact, a Spd-rich diet postpones age-related phenomena, such as the progressive decline of locomotor activity in flies [52]. In this variety of model organisms, Spd was found to suppress several aging-associated parameters, such as overproduction of ROS and the level of necrotic cell death [51]. Recently, Spd has been of great interest in the prevention or treatment of muscle diseases, since it may play a role in skeletal muscle atrophy/hypertrophy [53]. Aging brings a loss of skeletal muscle, and it has been shown that Spd cellular concentrations decrease during age progression [51]. Autophagy contributes to age-related degeneration processes, since it has been proven to decrease with aging. In physiological conditions, autophagy has a critical role, acting as a cell housekeeper by degrading damaged or unnecessary organelles and allowing the recovery of metabolites under nutrient starvation [54,55]. Deregulated autophagy contributes to neurodegenerative disorders, as well as liver, heart, and muscle diseases [56]. Variations in autophagic flux have been demonstrated to affect muscle homeostasis and body metabolic state [56]. Different studies have analyzed the correlation between aging, Spd, and autophagy and led to the identification of Spd as a strong and specific inducer of autophagy [56]. Through the autophagy mechanism, Spd extends lifespan by triggering epigenetic deacetylation of histone H3 through inhibition of histone acetyltransferases, suppressing oxidative stress and necrosis [51]. This mechanism provides protection from the aging process for several tissues, including heart, brain, and skeletal muscle, thereby endorsing longevity [1]. In an aged population, regular and proper exercise is used as a stimulus for muscle adaptation, attenuating the loss of skeletal muscle [57]. Autophagy attenuation that occurs with aging has been shown to be reduced with exercise training, thus endurance exercise enhances autophagic signaling in aged mice [1]. The coupled use of a specific autophagic inducer such as Spd and regular exercise could activate autophagy, establishing a correct health level for the maintenance of skeletal muscle [1].

Extended bed rest or post-surgery immobilization, resulting in skeletal muscle unloading, leads to skeletal muscle atrophy. In addition, exposure to a microgravity environment during extended space flights brings skeletal muscle disuse/atrophy similar to what is observed in prolonged bed rest or post-surgery recovery [53]. Abukhalaf et al. [53] reported that skeletal muscle polyamine levels appear to be influenced by microgravity (hind limb suspension) in a fiber-type-specific manner. Unloading-induced atrophy was accompanied by a dramatic decrease in Spd level (68%) in slow-twitch (type I) muscle fibers, but a slight increase (14%) in fast-twitch (type II) ones, when comparing unloaded and control animals [53]. No significant changes were observed in Spm levels in either type of muscle fibers [53]. On the other hand, individuals suffering from myasthenia gravis display high Spd and Spm levels, associated with muscle weakness and severe atrophy [58]. Further contrasting results were obtained by Bongers et al. [30], who did not detect any change of Spd levels in limb immobilization, suggesting that enzymes other than SMOX or transporters are able to maintain Spd levels when SMOX activity is reduced. Taking into account all these works, it can be seen that the direct role of polyamines, and in particular Spd, needs to be clarified in detail, but it is necessary to refer to the specific type of muscle fiber in light of the results obtained by Abukhalaf et al. [53].

A frequently used model to induce aging-related damage in vivo is administration of D-galactose (D-gal) [59], which causes accumulation of ROS with final oxidative stress [60].

Excessive apoptosis and deficient autophagy may cause skeletal muscle atrophy due to pathological events or the aging process, thus promoting cell death and disease progression [1]. Recently, AMP-activated protein kinase (AMPK) has been found to phosphorylate forkhead box O3 (FOXO3a) on Ser588 [61]. The FOXO3a signaling pathway induces the transcriptional activation of autophagy-related genes that oversee protein degradation. FOXO3a was found to be necessary for the reduction of skeletal muscle atrophy induced by D-gal and to maintain proliferation in aging skeletal muscle cells by Spd and exercise. These results suggest that exercise and Spd may share mediators that act on similar pathways in varying degrees, generating a synergistic effect for delaying skeletal muscle senescence [1]. Therefore, Spd, by activating the AMPK-dependent autophagy pathway, may decrease endoplasmic reticulum stress and reduce apoptosis [62].

Skeletal muscle atrophy is a state that not only characterizes a physiological condition in the aged population or in extended bed rest, but also is present in several pathologies with different etiologies. 

Spermidine was found to ameliorate myopathic defects in the animal model of Ullrich congenital muscular dystrophy (UCMD) and Bethlem myopathy (BM) (*col6a1^−/−^* mice) by reactivating autophagy in skeletal muscle [56]. In this model, there is overactivation of AKT, which causes defective autophagy by activating the mechanistic target of rapamycin, which inhibits the transcription of genes under FOXO [56]. Ineffective autophagy brings an accumulation of damaging organelles in the myofibers, which degenerate with time. However, this process is reversible through dietary and pharmacological approaches [63]. The beneficial effects of Spd administration in *col6a1^−/−^* mice are linked to its ability to reactivate autophagic flux [64], as demonstrated by a significant increase of LC3B and autophagosome formation [56]. Spermidine seems to act on AKT [56]. AKT kinase negatively regulates, by phosphorylation, the activity of FOXO transcriptional factors. Low levels of phosphorylated AKT activate FOXO [65]. Spermidine is able to reduce AKT phosphorylation, which triggers the translocation of FOXO transcriptional factors into the nucleus, promoting autophagy [56]. BM and UCMD patients display respiratory insufficiency due to a loss of diaphragm function [66], which is also the most affected muscle in *col6a1^−/−^* mice, where it shows a high incidence of apoptotic myofibers [63]. Spermidine was able to rescue the aspects of both BM and UCMD.

**Figure 5:**
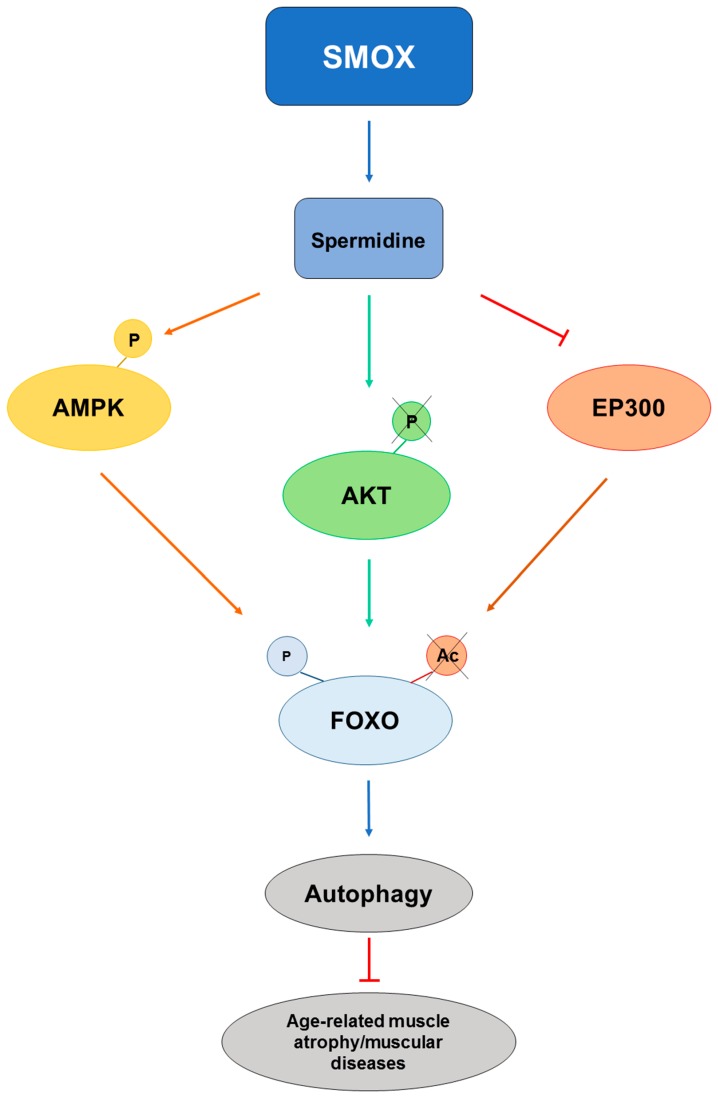
Schematic representation of spermidine effects of on forkhead box (FOXO) activity. The effect of spermidine on the attenuation of age-related skeletal muscle atrophy and diseases, through regulating autophagy via 5′ AMP-activated protein kinase (AMPK)/Protein Kinase B (AKT)/E1A binding protein p300 (EP300)-FOXO signal pathways.

FOXO proteins are key transcription factors regulated by different post-translational modifications, and among these, inhibitory acetylation by the histone acetyltransferase EP300 has been described in detail [67]. Since Spd also acts as a histone acetyltransferase inhibitor, and one of its known targets is EP300 [68], it is probable that Spd controls FOXO activity at multiple levels and determines a permissive condition for its activity by the action of AMPK, AKT, and EP300 (Figure 5). 

## 7. Polyamines and Muscle Diseases

The involvement of Spd, and of PAs in general, has also been demonstrated in amyotrophic lateral sclerosis (ALS) and Duchenne muscular dystrophy. Results from ALS patients and SOD1^G93A^ mice, an animal model for ALS pathology, showed deregulated PA levels in plasma, skeletal muscle, and cerebral cortex. The many roles of PAs include ALS-relevant processes such as protection against stress induced by ROS [69], modulation of glutamate ion channel receptors [70], and induction of autophagy [51]. Similarly, muscular fibrosis after denervation displays a constant rise in PA concentration [71].

Duchenne muscular dystrophy is a neuromuscular disease caused by mutation(s) in the dystrophin gene. It is characterized by skeletal muscle and cardiopulmonary complications, resulting in shorter life expectancy. The mouse model that better represents this disease is a double-mutant mouse (*dmd*/*utrn* double mutant (*mdx-dm*)), where both dystrophin and utrophin genes are mutated [72]. It is hypothesized that the rapid deterioration of muscles is correlated to reduced action of androgens [73]. In a recent study, GTx-026 a nonsteroidal selective-androgen receptor modulator that selectively builds muscle and bone, was used to treat *mdx-dm* mice, and it was able to increase muscle mass, function, and survival [72]. While GTx-026 failed to reverse the genes altered by dystrophin knockdown, it regulated the expression of several genes of the PA pathway. Interestingly, GTx-026 significantly increased SMOX by two- to threefold. Moreover, genes belonging to the Spd pathway were also upregulated by GTx-026, confirming the crucial role of Spd in enhancing cellular lifespan and proposing SMOX as a key gene in muscle diseases [72].

## 8. The Transgenic Mouse Line: *Total-Smox*

**Figure 6:**
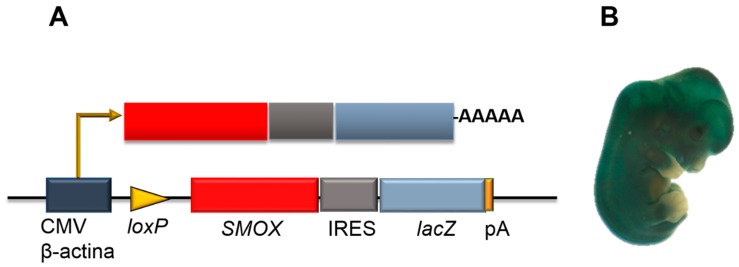
*Total-Smox* mouse line. (**A**) Scheme of the genetic construct of the *Total-Smox* mouse line upon recombination of the loxP sites by Cre recombinase. The β-actin/Cytomegalovirus (CMV) fusion promoter drives the ubiquitous expression of the *SMOX* gene and the *lacZ* reporter gene. IRES: internal ribosome entry site. (**B**) LacZ staining of *Total-Smox* embryo.

Spermine oxidase is a significant positive regulator of muscle gene expression and fiber size. Recently, a Cre/loxP-based double transgenic mouse line overexpressing the Smox gene in all organs was engineered and named Total-Smox. Transgenic Green Fluorescent Protein (GFP)-Smox (formerly JoSMOX) mice described by Cervelli et al. [6] were crossed with Total-CRE mice reported by [74], to obtain the Total-Smox genetic line (Figure 6). This new experimental model was further genetically stabilized by back-crossing Total-Smox mice 10 times with C57BL/6 mice. SMOX overexpression in transgenic individuals can be detected in all tissues by β-Galoctiside (3-Gal) staining (Figure 6) and reverse-transcriptase/PCR analysis. As expected, SMOX enzyme activity was higher in all the organs of Total-Smox mice in comparison to their syngenic littermates, including skeletal muscle and heart, as demonstrated by [75].

In the literature, it is known that muscle diseases and aging-related pathologies display high oxidative stress by accumulating H_2_O_2_ in the muscles [76]. In the Total-Smox mouse model, chronic H_2_O_2_ production due to SMOX overexpression leads to an imbalanced cellular redox state in both types of muscle tissue. Skeletal muscle displays lower oxidative damage compared to the heart, evoking a different redox adaptation through upregulation of the enzymatic antioxidant system [75]. This tissue shows a significant decline in the ratio of GSH reduced and oxidized due to a decrease in the total amount of GSH. SMOX overexpression increases glutathione S-transferase activity, since it is an enzyme induced under oxidative stress and is responsible for the detoxification of molecules through the formation of S-conjugates [75]. SMOX overexpression in the skeletal muscle of Total-Smox mice also causes a concomitant increase of catalase, another detoxifying enzyme, indicating that there is a counteracting action against the high H_2_O_2_ production. Moreover, the amount of Spd has been found to be elevated in Total-Smox mice compared to control animals, while the levels of Put and Spm were not altered. Spermidine has been proven to be essential in muscle physiology by promoting autophagy, counteracting aging, and extending lifespan [1].

Considering the important role of Spd in many pathologies and that Spd increases in the *Total*-*Smox* mice model while no changes are reported for the other PAs, it would be interesting to cross this mouse line with animal models of different diseases such as Ullrich congenital muscular dystrophy and Bethlem myopathy (*col6a1^−/−^* mice), amyotrophic lateral sclerosis (SOD1^G93A^ mice), and Duchenne muscular dystrophy (*mdx-dm* mice), to evaluate the possibility of rescuing the phenotype. 

## 9. Conclusions and Future Perspectives

This review points out that not only does SMOX take part in cancer and neurological disorders, but it is also involved in skeletal muscle pathophysiology. New evidence for the physiological roles of the PA pathway in atrophy could represent a major area for future research [77]. The prevalence rate of sarcopenia is up to 33% in elderly people, and the number is expected to increase due to lifestyle, dietary habits, and aging [78]. It has been known that PA levels and related enzyme activities decline during aging; however, until recently, it was not clear how alterations in PA metabolism could affect the aging process [79]. Several experiments have highlighted the role of Spd as an autophagy inducer that ameliorates age-related muscle atrophy [80]. Different mouse models of disease have brought new insights as to the possibility of Spd as a therapeutic food option. This approach could be used to design innovative treatments, with Spd alone or in combination with other approaches, leading to clinical trials. In perspective, this nutraceutical-based, autophagy-inducing approach could be applied to different therapies, and could also be extended to other inherited muscle pathologies involving defective activity of the autophagy machinery. Of note, Spd is the reaction product of SMOX, which in turn has been demonstrated to help in maintaining muscle mass and counteracting the expression of genes that promote muscle atrophy. Understanding how the SMOX/p21 axis and its product Spd influence muscle gene expression and how they are linked to autophagy and muscular atrophy during aging is a new field of investigation. Considering that SMOX is highly expressed in skeletal muscle and regulates the amount of PAs, it can become a key gene to target for treatment of diseases such as Duchenne muscular dystrophy.

In perspective, *Total*-*Smox* mice could be considered as a valuable genetic animal model to investigate the role of SMOX and Spd in muscular physiology, shedding light on new therapies to test in several muscle diseases.
